# Exploring the impact of violence in video games

**DOI:** 10.7554/eLife.94949

**Published:** 2024-01-16

**Authors:** Nicolas Roy, Michel-Pierre Coll

**Affiliations:** 1 École de Psychologie, Université Laval Quebec City Canada; 2 Centre interdisciplinaire de recherche en réadaptation et intégration sociale Québec Canada

**Keywords:** violent video games, empathy, emotional reactivity, fmri, desensitization, Human

## Abstract

Playing a violent game for a few weeks did not alter neural and behavioral responses to the pain of others in inexperienced male gamers.

**Related research article** Lengersdorff LL, Wagner IC, Mittmann G, Sastre-Yagüe D, Lüttig A, Olsson A, Petrovic P, Lamm C. 2023. Neuroimaging and behavioral evidence that violent video games exert no negative effect on human empathy for pain and emotional reactivity to violence. *eLife* **12**:e84951. doi: 10.7554/eLife.84951.

The release of *Grand Theft Auto III* in 2001 marked a turning point in the public discussion around video games (McLaughlin, 2008). With graphics more lifelike than ever, the game allowed players to act as criminals free to roam a city and commit senseless acts of violence against its population. Inevitably, social and research questions were raised regarding how this type of media could impact social and emotional wellbeing, in particular in the young men who form most of the gaming community. How would playing highly realistic games that allow the gratuitous murder and exploitation of others impact their psychological functioning?

This question has been notoriously difficult to answer scientifically, and it remains harshly debated (Devilly et al., 2023; Bushman and Anderson, 2021). Indeed, both real world observations and lab-based experiments have limitations when trying to assess how gaming may impact emotional, neural and behavioral mechanisms. Observational studies, which rely on measuring these processes without influencing them, are confounded by the fact that violent games tend to attract individuals who already have specific personality and social profiles (Braun et al., 2016). In contrast, experimental work is restricted by practical considerations; in the laboratory, participants can only be exposed to games for short periods, for example. It has also been hindered by inadequate study design, with experiments featuring control conditions that fail to effectively isolate violent content, or recruiting participants who have extensive experience with violent games. Now, in eLife, Claus Lamm and colleagues at the University of Vienna and Karolinska Institutet – including Lukas Lengersdorff as first author – report having designed an experimental study that overcomes many of these limitations (Lengersdorff et al., 2023).

The team recruited 89 young men with little gaming experience and no previous exposure to *Grand Theft Auto V* (GTA V). Half the participants were assigned to play a normal version of the game and incentivized to kill as many people as possible; the other half accessed a modified version of GTA V devoid of all violent content and got rewarded for taking pictures of other characters. Both groups played for seven hours over two weeks in a supervised lab setting. In addition, the participants’ neural and behavioral responses to images of people in pain or in emotionally charged situations were measured at the start and the end of the study, with Lengersdorff et al. using well-established fMRI and behavioral approaches to measure empathy and emotional reactivity (Singer et al., 2004).

The results showed that the two groups showed no neural or behavioural differences in the response to the distress of others. Additionally, statistical analyses using Bayesian techniques further suggested that playing a violent or non-violent version of the game had no effect on empathy for pain or emotional reactivity. Overall, these analyses provide solid evidence that men with little gaming experience do not get desensitised to the emotions of others after using violent games for a short period.

As Lengersdorff et al. highlight, however, the tightly controlled nature of this lab-based study may limit the generalization of the findings to other demographics or more typical video game use. For example, it remains unclear if the same results would emerge in people of different genders or those already drawn to violent games. The relatively short gaming period used in the study may also not accurately represent the habits of habitual gamers, who, on average, engage in gaming for about 16 hours a week over many years (Clement, 2021).

Nevertheless, the findings of Lengersdorff et al. align with accumulating evidence that playing violent video games has, by itself, little to no substantial impact on emotional and social functioning (Ferguson et al., 2020; Kühn et al., 2019). Although these studies do not imply that consuming this type of media is never problematic, they do suggest it can be a part of a healthy lifestyle. More than two decades after the release of GTA III, it may be time to move beyond the moral panic and the simplistic assumption that violent video games are inherently damaging. Future scientific research should aim to delineate the specific circumstances under which this type of media may contribute to psychological distress and antisocial behaviours without neglecting the idea that it might, in some cases, offer positive benefits to players (Etchells, 2019).
